# Blood–Brain Network-Based Polygenic Risk Scores Reveal Biomarker Signatures and the Progression of Alzheimer’s Disease

**DOI:** 10.3390/jcm15082885

**Published:** 2026-04-10

**Authors:** Daniel Goldstein, Nathan Sahelijo, Dhawal Priyadarshi, Rebecca Panitch, Kwangsik Nho, Lindsay A. Farrer, Thor D. Stein, Gyungah R. Jun

**Affiliations:** 1 Biomedical Genetics Section, Department of Medicine, Boston University Chobanian & Avedisian School of Medicine, Boston, MA 02118, USA; 2 Bioinformatics Program, Faculty of Computing & Data Sciences, Boston University, Boston, MA 02215, USA; 3 Department of Radiology and Imaging Sciences and Medical and Molecular Genetics, Indiana University School of Medicine, Indianapolis, IN 46202, USA; 4 Indiana Alzheimer’s Disease Research Center, Indiana University School of Medicine, Indianapolis, IN 46202, USA; 5 Center for Computational Biology and Bioinformatics, Indiana University School of Medicine, Indianapolis, IN 46202, USA; 6 Boston University Alzheimer’s Disease Research Center, Boston University Chobanian & Avedisian School of Medicine, Boston, MA 02118, USA; 7 Department of Neurology, Boston University Chobanian & Avedisian School of Medicine, Boston, MA 02118, USA; 8 Department of Epidemiology, Boston University School of Public Health, 715 Albany Street, Boston, MA 02118, USA; 9 Department of Biostatistics, Boston University School of Public Health, 715 Albany Street, Boston, MA 02118, USA; 10 Department of Ophthalmology, Boston University Chobanian & Avedisian School of Medicine, Boston, MA 02118, USA; 11 Department of Pathology & Laboratory Medicine, Boston University Chobanian & Avedisian School of Medicine, Boston, MA 02118, USA; 12 Veterans Affairs Bedford Healthcare System, Bedford, MA 01730, USA; 13 Veterans Affairs Boston Healthcare Center, Boston, MA 02130, USA

**Keywords:** Alzheimer’s disease, co-expression network, preserved network between blood and brain, network-based polygenic risk score, survival analysis, disease incidence, gene profiling, HLA locus

## Abstract

**Background:** Polygenic risk scores for Alzheimer’s disease (AD), organized by gene networks shared between the blood and brain, may provide insights into underlying disease mechanisms common to both tissues. **Methods:** We derived a blood–brain network-based polygenic risk score (nbPRS) from AD-associated genetic variants for three blood-brain networks, selected by the preservation of blood and brain gene co-expression networks, and AD association. Participants from the Alzheimer’s Disease Neuroimaging Initiative (ADNI, n = 1109), Framingham Heart Study (FHS, n = 8310), the Religious Orders Study Memory Aging Project (ROSMAP, n = 1215), and Mount Sinai Brain Bank (MSBB, n = 323) were stratified into low- and high-nbPRS subgroups, then profiled using longitudinal and cross-sectional data. We compared the conversion from normal cognition to AD between nbPRS subgroups. Genes differentially expressed among low- and high-nbPRS individuals were profiled with classical neuropathological markers and we investigated potential biologically relevant pathways for the genes significantly expressed in high-risk individuals. **Results:** Individuals with high nbPRS in three AD-associated networks (M2, M6, M14) demonstrated significant impairment in executive function and memory performance, whereas high-risk individuals in networks M2 and M14 had significantly reduced hippocampal volume. We observed high-risk individuals in M2 and M14 developed AD at twice the rate of low-risk individuals in these networks. *HLA* genes were differentially expressed with transcriptome-wide significance among low- and high-nbPRS individuals in M14 and associated with neuroinflammatory and tau pathology. **Conclusions:** Polygenic risk scores derived from blood and brain networks can differentiate individuals with a high risk of AD conversion.

## 1. Background

Alzheimer’s disease (AD) is a complex neurodegenerative disorder that presents with impairment in cognitive domains, such as memory, language, executive function, and visuospatial function. There are 6.5 million people aged 65 or older estimated to be living with AD in the US [1]. Hundreds of clinical trials for AD treatments have been withdrawn, suspended, or terminated within the last two decades [2,3,4]. A major contributing factor for the high failure rate of AD trials is the clinical and neuropathological heterogeneity in clinical trial participants [5]. Differences in cognitive symptoms, neuropathological traits, age at onset, and rate of progression among AD patients indicate the presence of different AD subtypes [6,7,8].

The polygenic risk score (PRS) is a metric used to assess an individual’s disease risk based on the genetic burden on the disease. The PRS has demonstrated clinical utility as a predictor for patient susceptibility to several heterogeneous diseases, including coronary artery disease [9,10,11], type 2 diabetes [10,11,12,13], and AD [14,15,16,17]. However, many of these studies have computed PRSs using genome-wide genetic markers (genome-wide PRS), which do not inform distinct biological mechanisms underlying the progression of heterogeneous diseases. Recent studies illustrated PRSs derived from networks of biologically connected genes such as type 2 diabetes [18], Parkinson’s disease [19], and AD [20,21,22] subtypes, providing biological insights into disease heterogeneity. A recent study demonstrated that the genome-wide PRS is useful to predict overall disease risk, while network-based PRSs successfully explain clinical (cognitive domains) heterogeneity and distinct brain atrophy patterns [20].

Blood-based biomarkers for AD are emerging as less invasive tools to screen individuals at risk to identify those who may have pre-clinical pathological changes associated with disease, including the accumulation of amyloid-β concentrated in senile plaques and neurofibrillary tangles resulting from the misprocessing of tau protein in the brain. Although there is extensive research on blood-based biomarkers for AD diagnosis, AD-associated transcriptome profiles have not been thoroughly investigated in the blood and brain from the same individuals. In our previous work, we revealed co-expression gene networks preserved in blood and brain transcriptome profiles significantly associated with AD and immune-related pathways [23].

In this study, we developed a novel approach leveraging network-based polygenetic risk scores (nbPRSs) using selected AD-associated variants within co-expressed gene networks preserved between blood and brain tissues, applied to individuals with normal or mildly impaired cognition who were followed longitudinally. Using these nbPRSs, we defined low- and high-nbPRS individuals (nbPRS risk status) and identified differentially expressed genes associated with each risk status to gain insights into the biological pathways through which these shared blood–brain networks operate. This will facilitate the early detection of AD-associated dysfunction in blood and support the development of more targeted therapeutic interventions for AD.

## 2. Materials and Methods

### 2.1. Data Sources for Co-Expression Networks Shared Between the Blood and Brain

**Figure 1:**
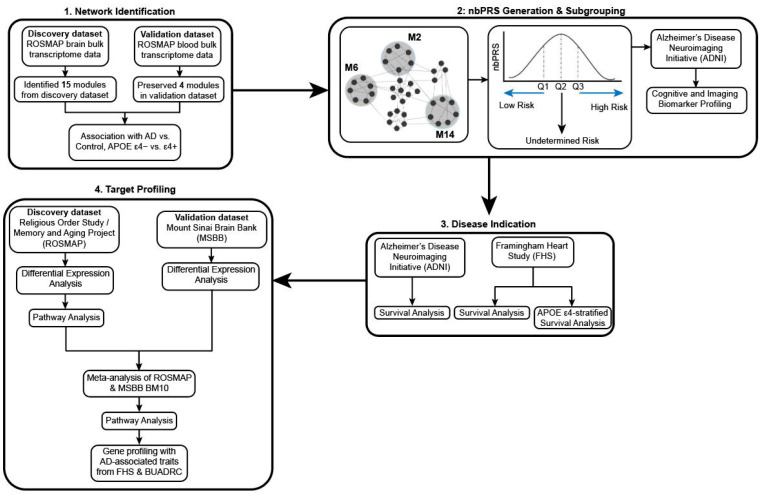
Study design diagram separated into four parts. Part 1: co-expression networks were identified with ROSMAP brain data and validated with ROSMAP blood data to find preserved blood brain networks. Part 2: nbPRSs were generated from these preserved networks and subjects from ADNI, FHS, ROSMAP, and MSBB were stratified into high- and low-nbPRS subgroups based on their risk score. Part 3: We assessed the predictive values of blood-brain network-preserved nbPRS for the rate of conversion from CN to AD. Part 4: Analysis of differential expression between low- and high-nbPRS subgroups and profile differentially expressed genes with known AD-associated neuropathological traits.

This study assessed the association of network-based polygenic risk scores (nbPRSs) from co-expressed gene networks preserved between the blood and brain with previously defined cognitive subtypes [23] and AD incidence. Panitch et al. (2022) evaluated these preserved networks [23] in relation to *APOE* genotypes (Figure 1, Part 1). For each preserved network, we computed a nbPRS in each of the four datasets, and participants were stratified into low- and high-nbPRS subgroups using the first and fourth quartiles of the nbPRS distribution (Figure 1, Part 2). Subsequently, we compared differential AD progression rates between these subgroups (Figure 1, Part 3). Finally, we explored the links between these differentially expressed genes and known AD-related cognitive and neuropathological traits (Figure 1, Part 4).

Bulk RNA sequencing data derived from dorsolateral prefrontal cortex tissue and blood donated by Religious Orders Study (ROS) and Rush Memory and Aging Project (MAP) participants were obtained from the CommonMind Consortium portal (http://www.synapse.org). The cohort included 576 individuals (346 clinically diagnosed AD cases and 230 controls) with data from autopsied brains, a subset of whom (n = 141; 85 clinically diagnosed AD cases and 56 controls) also provided data from blood. Procedures for processing and quality control of these data are described elsewhere [23]. Previously, four co-expression gene networks preserved in the blood and brain were identified using weighted gene correlation network analysis (WGCNA) using bulk RNA sequencing data from the ROSMAP study [23,24]. Briefly, network preservation in the blood and brain was evaluated using the module Preservation function in WGCNA, with a preserved blood–brain network defined with Z_summary_ score > 5 [23,25]. Eigengenes were derived from the first principal component for each module and served as representative values of gene expression in a given network [26]. This study evaluated the association of expression of eigengenes in blood and clinical diagnosis (AD vs. control) using a linear regression model adjusting for age at exam and sex. We nominated AD-associated networks with *p* < 0.05 for subsequent analyses.

### 2.2. Data Sources for Polygenic Risk Score Generation

Genetic data from four genome-wide association study (GWAS) datasets were used to generate nbPRSs. The Alzheimer’s Disease Neuroimaging Initiative (ADNI) is a longitudinal study with clinical, imaging, genetic, and biomarker examinations to monitor progression from normal cognitive function to AD or AD-related dementia (ADRD). Genetic data and AD-related domain-specific cognitive test scores of ADNI participants were obtained from the LONI website (http://adni.loni.usc.edu). Details of quality control (QC), imputation, and population substructure analysis procedures using GWAS data are described elsewhere [20]. At the baseline exam, there were 315 cognitively normal (CN), 610 mild cognitive impairment, and 197 AD participants. Three to five years following the baseline exam, the number of AD cases increased to 456 participants (Supplementary Table S1). Among CN individuals at baseline, 35 individuals were converted from CN to MCI and 15 individuals from CN to AD during follow-up examinations.

The Framingham Heart Study (FHS), established in 1948 to study risk factors for cardiovascular disease, is a multi-generational population-based longitudinal study of health. All 8310 individuals who have participated in neuropsychological examinations enter the FHS with CN function at baseline; however, after 42 years of monitoring up to 13 neuropsychological evaluations, 310 participants developed MCI and 540 developed AD (Supplementary Table S1). A consensus AD diagnosis was established by a panel of clinicians, and sub-classified as either AD without stroke, AD with stroke, or mixed AD (AD with vascular disease).

For nbPRS generation, we used GWAS data from the full set of 2089 ROSMAP participants and 323 individuals in the Mount Sinai Brain Bank (MSBB) obtained from the CommonMind Consortium portal (http://www.synapse.org) [27]. The ROS and MAP studies are prospective and longitudinal clinical cohort studies investigating aging and AD [28,29]. Both studies were managed by a single supervisor and trainer and data collected by one team at the item level to facilitate integrated analyses [30]. Clinical diagnoses were established as previously described in detail [31,32,33]. Neuropathological data were obtained from 440 cognitively normal (CN) controls and 775 AD participants at their death (Supplementary Table S2). The MSSB participants included in this study were chosen following strict selection criteria to represent a range of dementia severity [34]. Cognitive status of these participants was evaluated using the clinical dementia rating (CDR) scale [35], and neuropathological assessments followed the guidelines from the Consortium to Establish a Registry for Alzheimer’s Disease (CERAD) protocol [35,36]. The MSSB study contained 71 AD and 252 CN subjects (Supplementary Table S2).

### 2.3. Generating Network Risk Status Using Network-Based Polygenic Risk Scores

For each blood–brain co-expression network, we computed a nbPRS using GWAS data for each participant separately in the ADNI, FHS, ROSMAP, and MSBB. Each nbPRS was calculated using effect sizes of single nucleotide polymorphisms (SNPs) located within the coding region (±20 k bases) of network genes and significantly associated with AD status (*p* < 10^−3^) [37]. SNPs with minor allele frequency < 1%, low imputation quality (R^2^ < 0.4), and highly correlated (R^2^ > 0.5) were excluded [37]. To characterize individuals with the lowest and highest AD risk, we investigated three definitions of a binary risk status for each network: (1) median stratification, (2) quartile stratification, and (3) tertile stratification. In median stratification, individuals were divided into risk subgroups by the median of the nbPRS across the population. In quartile and tertile stratification, individuals in the most extreme quantiles were used for comparison and all individuals in intermediary quartiles were excluded from the analysis; therefore, we defined individuals in the first quantile as low-nbPRS risk and individuals in the last quantile as high-nbPRS risk, which refer to the fourth and tenth quantiles, respectively. This stratification method enables increased contrast at the risk of reducing statistical power.

### 2.4. Association of Cognitive and Brain Imaging Traits for Network Risk Status in ADNI Patients

We evaluated the association of cognitive test (composite executive function and composite memory) scores and brain imaging measures, including global amyloid level, hippocampal volume, temporal fluorodeoxyglucose level (FDG), and entorhinal cortex thickness with network risk status as a binary outcome in ADNI participants using logistic regression models including covariates for age at last exam and sex. Models testing association with hippocampal volume or entorhinal cortex was further adjusted for intracranial volume and magnetic field strength.

### 2.5. Investigating Differential AD Progression for Network Risk Status in ADNI and FHS Patients

We compared the conversion rate from CN to incident AD between individuals in the low- and high-nbPRS subgroups in the ADNI and FHS datasets using Cox proportional hazards models implemented in the *survminer* package in R version 4.2.0 [38]. In the FHS, we evaluated AD conversion in nbPRS subgroups stratified by median, quartile, and tertile. Individuals with AD and age > 75 at baseline or age < 65 at last exam were excluded. Models included covariates for age at baseline, sex, *APOE* genotype, low-density lipoprotein (LDL) level, and fasting blood glucose (glucose). Analyses of the FHS dataset included a term for family structure as an additional covariate. The difference of the incidence rate for each network risk status was expressed as a hazards ratio (HR), which describes the likelihood of participants in the high-nbPRS group developing AD compared to those in the low-nbPRS group. We evaluated the effect of the *APOE* ε4 allele on the conversion rate by analyzing Cox proportional hazards models separately in *APOE* ε4 carriers and non-carriers and including the *APOE* genotype as a covariate.

### 2.6. Differential Expression Analysis for Network Risk Status in ROSMAP and MSBB Patients

We investigated the differential expression of genes for network risk status using 595 ROSMAP participants with both GWAS and bulk RNA sequencing data. We applied quality control procedures and normalized the bulk ROSMAP RNA sequencing (RNA-seq) data as previously described [23]. RNA sequencing data from the prefrontal cortex area (Brodmann area 10; BM10) of 170 MSBB brains were obtained from the CommonMind Consortium portal (http://www.synapse.org) [27]. Quality control procedures applied to MSBB RNA sequencing data, which were normalized after adjustment for sex, race, age, RNA integrity number (RIN), postmortem interval (PMI), RNA batch, exonic rate, and rRNA rate, were reported elsewhere [34]. We conducted transcriptome-wide differential expression analysis for each network using network risk status as a binary outcome using a regression model implemented in LIMMA software in R [39,40], including sex, age at death, PMI, RNA batch, and RIN as covariates. Results from the ROSMAP and MSBB datasets were combined by meta-analysis using METAL software with the sample-weighted option [41]. We evaluated the cell-type-specific gene expression profiles of top-ranked genes using ROSMAP single-cell RNA-seq data previously reported [23].

### 2.7. Pathway Analysis Using Differentially Expressed Genes for Network Risk Status

We conducted pathway enrichment analysis for significantly differentially expressed genes with *p* < 0.05 for each network risk status using the Gene Ontology Biological Process (GOBP), Gene Ontology Molecular Process (GOMF), and KEGG pathway databases within the *enrichR* package in R [42]. Enrichment analysis was conducted separately for upregulated and downregulated genes. We created subnetworks of the top-ranked network using Ingenuity Pathway Analysis (IPA) software (Qiagen Inc., Valencia, CA, USA) that were seeded with differentially expressed genes with *p* < 0.05 for network risk status in the combined ROSMAP and MSBB meta-analyzed dataset (seed genes). These subnetworks were further analyzed using GOBP, GOMF, and KEGG databases using the *enrichR* option in R to identify biologically relevant pathways.

### 2.8. Association of Expression of Top-Ranked Genes with AD-Related Traits in FHS and BUADRC Brains

We evaluated associations of expression of seed genes in the selected subnetwork from the top-ranked network with pathological AD diagnosis and several AD-related traits measured in the prefrontal cortex area of autopsied brains from the FHS and Boston University Alzheimer’s Disease Research Center (FHS/BUADRC) [43]. Neurofibrillary tangles were assessed by Braak staging, and neuritic plaques were quantified by CERAD scores. Associations were also assessed with other tau-related measures, including anti-tau monoclonal antibody 8 (AT8), phosphorylated Tau at positions 181 (pTau181), 202 (pTau202), 231 (pTau231), and 396 (pTau396). Amyloid pathology-related measures contained anti-amyloid monoclonal antibody 4G8 (4G8) and amyloid-β proteins 40 (Aβ40) and 42 (Aβ42). Several neuroinflammatory molecules were also evaluated, including levels of ionized calcium binding adaptor molecule 1 (Iba1), CD68 protein, and complement proteins including C4a, C4b, and C1q. Neuropathological traits were rank-transformed after adjusting for age at death and sex.

## 3. Results

### 3.1. AD-Associated Co-Expression Networks and Distribution of nbPRSs

In the previously published study [23], we identified four co-expressed gene networks, blue (M2), green (M6), green–yellow (M7), and turquoise (M14), which were preserved in blood and brain samples with a Z_summary_ score > 5, with a number of network genes ranging from 230 genes in M7 to 4169 genes in M14 (Supplementary Table S3). We excluded the M7 network for our analysis, since this network was not associated with AD (Supplementary Table S3). Expression of genes in the M7 network was increased in *APOE* ε4 carriers compared to *APOE* ε4 non-carriers as reported by Panitch et al. [23].

Of the M2, M6, and M14 networks, the M14 network showed the strongest statistical association with AD status (r = −0.11, *p* = 6.0 × 10^−3^). The distributions of nbPRSs approximated a normal distribution across all three networks (M2, M6, and M14) in all datasets (Supplementary Figure S1), with comparable mean nbPRS values across studies (Supplementary Table S4). After stratifying individuals into high- and low-nbPRS subgroups, the numbers of individuals in each subgroup were approximately equal across the four studies (Table 1). As expected, individuals with a high genetic risk had higher average nbPRSs than those with a low risk.

The number (N) of subjects stratified into low-risk and high-risk groups based on network-based polygenic risk scores (nbPRSs) using the first and fourth quartiles of nbPRSs, respectively, were analyzed. We excluded subjects between the first and the fourth quartiles of the nbPRS distribution. Mean and standard deviation (SD) were provided for nbPRS and age at last exam or age at death.

### 3.2. Association of Cognitive and Imaging Traits with Network Risk Status in ADNI Patients

**Figure 2:**
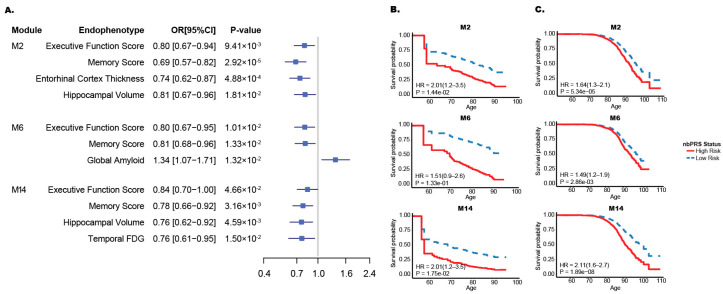
Effect of network risk status on biomarker and AD progression. (**A**) Comparison of endophenotypes based on the risk status of M2, M6, and M14 networks in ADNI patients. Network risk status was dichotomized into low- and high-risk subgroups using the first and fourth quartiles of the network-based polygenic risk score (nbPRS) distribution. Executive function and memory composite scores represent combined performance across multiple cognitive tests within their respective domains. OR: odds ratio; CI: confidence interval. Cox proportional hazard models comparing low- and high-nbPRS subgroups in ADNI (**B**) and FHS (**C**) patients.

Executive function and memory domain scores in ADNI patients were significantly associated with network risk status for all three networks (Figure 2A). Association of a lower memory domain score for the M2 risk status was most significantly associated with a low-risk status with an odds ratio (OR) of 0.69 (*p* = 2.92 × 10^−5^). Hippocampal volume was significantly reduced in high-risk compared with low-risk participants for the M2 (OR = 0.81, *p* = 0.019) and M14 (OR = 0.76, *p* = 0.0046) networks. Amyloid pathology measured by PET scan (global amyloid) was significantly increased among individuals with a high-risk status for the M6 network (*p* = 0.013).

### 3.3. Effect of Network Risk Status on AD Progression

In the ADNI sample, individuals with a high-nbPRS status had a higher AD conversion rate compared to those with a low-nbPRS status (*p* < 0.02) for the M2 and M14 networks (Figure 2B). The hazard ratios (HRs) for conversion to AD in ADNI patients were identical (HR = 2.01) for both networks (Table 2). In the FHS sample, the conversion rate to AD was significantly higher among high-nbPRS individuals in the M2, M6 and M14 networks (Figure 2C), with the strongest effect observed in the M14 network (HR = 2.11, *p* = 1.89 × 10^−8^; Table 2). Hazard ratios (HRs) using median or tertile cutoffs did not show consistent improvement over quartile stratification. Notably, quartile stratification yielded the most robust HR for the M14 network (Supplementary Table S5). Furthermore, because the tertile cutoff resulted in a smaller number of AD cases per group, we focused our primary analysis on the quartile stratification (Supplementary Table S6). The findings with the quartile stratification in FHS individuals remained significant even among persons lacking the *APOE* ε4 allele (Supplementary Table S7 and Supplementary Figure S2) or including the *APOE* genotype as a covariate in a non-stratified Cox proportional hazards model (Supplementary Table S8 and Supplementary Figure S3A). Similarly, LDL and fasting glucose levels had a minimal impact on AD onset in high-risk individuals (Supplementary Table S8 and Supplementary Figure S3B). We selected the M14 network for further analysis for transcriptome-wide differential expression analysis.

### 3.4. Differentially Expressed Genes for M14 Risk Status

**Figure 3:**
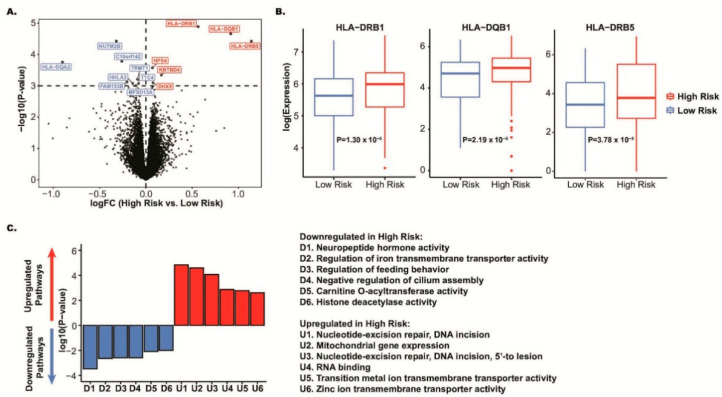
Differential gene expression analysis of the M14 network in ROSMAP. (**A**) Volcano plot displaying differentially expressed genes between low- and high-nbPRS subgroups, with log fold change (x-axis) plotted against -log *p*-value (y-axis). Genes with significantly increased (red, *p* < 0.001) and decreased (blue, *p* < 0.001) expressions in high-nbPRS groups are highlighted. (**B**) Expression comparison of the two most significantly differentially expressed genes between low- and high-nbPRS subgroups, with corresponding *p*-values provided. (**C**) Top gene ontology (GO) terms associated with significantly upregulation (red) and downregulation (blue) in high-nbPRS groups, ranked by *p*-value and plotted by expression direction.

At a significance threshold of *p* < 0.001 in the M14 network, we observed six upregulated and eight downregulated genes in the ROSMAP discovery sample (Figure 3A and Supplementary Table S9). The most significantly differentially expressed gene was in the *HLA* locus (*HLA-DRB1*: *p* = 1.30 × 10^−5^), where two additional genes in the *HLA* locus (*HLA-DQB1* and *HLA-DRB5*) were also significant with *p* < 10^−3^ (Figure 3B and Supplementary Table S9). *HLA-DRB5* had the greatest fold change in expression (logFC = 1.14) between high- and low-nbPRS individuals (Table 3), whereas *HLA-DQA2* had the greatest fold change of downregulated genes (logFC = −0.90; Figure 3A). The top three genes in the *HLA* locus (*HLA-DRB1*, *HLA-DQB1*, and *HLA-DRB5*) in the discovery study were confirmed in the MSBB replication sample at a nominal significance level (*p* < 0.05). Two of the *HLA* genes were differentially expressed at the transcriptome-wide significance level at *p* < 10^−6^ (*HLA-DRB1*: *p* = 1.77 × 10^−7^ and *HLA-DQB1*: *p* = 2.79 × 10^−7^) (Table 3). Cell-type expression profiling of differentially expressed genes (DEGs, *p* < 10^−3^) between AD and control cells revealed that top-ranked *HLA* genes within the M14 network were significantly differentially expressed in neurons but not in microglia (Supplementary Figure S4). Further examination of the linkage disequilibrium (LD) structure comprising SNPs with *p* < 10^−6^ on chromosome 6 from the M14 network nbPRS identified three potentially independent LD blocks contributing to the network’s polygenic risk (Supplementary Figure S5).

Pathways associated with upregulated genes with *p* < 0.05 in the M14 network included nucleotide excision repair, RNA binding, and transmembrane transporter activity (Figure 3C). Pathways associated with downregulated genes with *p* < 0.05 in the M14 network were enriched in significant pathways, including neuropeptide hormone activity, histone deacetylase activity, regulation of transmembrane transporter activity, feeding behavior, and cilium assembly (Figure 3C).

### 3.5. Identification of M14 Subnetwork and Its Associations with Gene Expression and Neuropathological Traits

**Figure 4:**
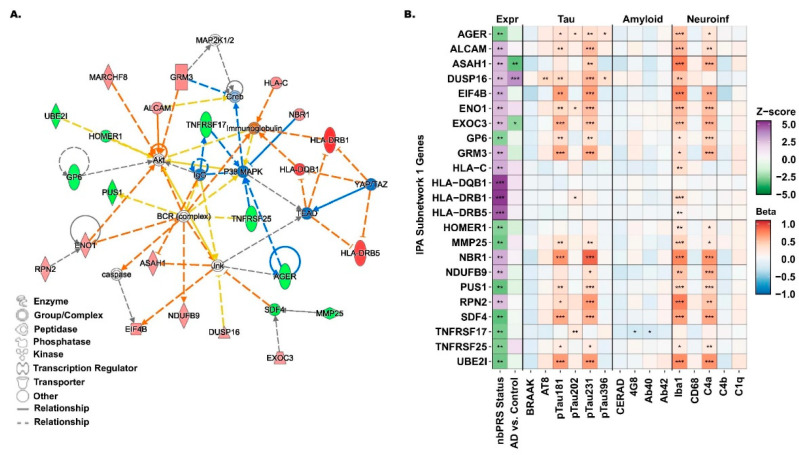
Network diagram and neuropathological associations of the subnetwork from M14. (**A**) Diagram of the M14 subnetwork with a score > 20, featuring genes from the *HLA* locus, including *HLA-DRB1* and *HLA-DQB1*, identified as the top differentially expressed genes between low- and high-nbPRS subgroups with *p* < 10^−6^ in the meta-analysis of ROSMAP and MSBB datasets. Genes are color-coded as upregulated (red), downregulated (green), predicted activation (orange), and predicted inhibition (blue) in the high-nbPRS subgroup. (**B**) Profile of subnetwork genes across multiple datasets. Expression profiles (Expr) for nbPRS status were derived from a meta-analysis of ROSMAP and MSBB brains. Expression analysis of AD (AD vs. control), tau pathology (Tau), amyloid pathology (Amyloid), and neuroinflammatory pathology (Neuroinf) were obtained from FHS brains. Statistical significance of gene associations is indicated as follows: *p* < 0.001 (***), 0.001 < *p* < 0.01 (**), and 0.01 < *p* < 0.05 (*).

We observed a biologically connected M14 subnetwork with 23 differentially expressed genes, including three from the *HLA* locus (*HLA-DRB1*, *HLA-DQB1*, and *HLA-DRB5*) with a predicted inhibition by TEAD and YAP/TAZ transcriptional regulators (Figure 4A). Other subnetwork genes, *PUS1*, *ENO1*, and *NDUFB9*, were predicted to be either activated or inhibited by the BCR complex (Figure 4A). *ALCAM*, *ENO1*, *GP6*, *GRM3*, *HOMER1*, *MARCHF8*, and *UBE21* genes were predicted as potential inhibitors or activators of the Akt signaling pathway, while *AGER*, *ASAH1*, *EIF4B*, *DUSP16*, and *SDF4* genes were predicted to activate or inhibit the Jnk pathway (Figure 4A). Expressions of *ASAH1*, *DUSP16*, and *EXOC3* genes from the subnetwork were significantly associated with both AD and M14 risk status (Figure 4B and Supplementary Table S9). Gene expression profiling of the subnetwork revealed strong associations with neuroinflammatory and tau pathology markers including Iba1, C4a, pTau181, and pTau231, but no association with amyloid pathology markers (Figure 4B and Supplementary Tables S11–S13). Expressions of all but two of the M14 subnetwork genes were positively associated with Iba1 (microglial) cellular density, most notably *RPN2*: *p* = 9.14 × 10^−7^, *HLA-DRB1*: *p* = 1.1 × 10^−6^, and *ASAH1*: *p* = 3.16 × 10^−6^ (Supplementary Table S13). Subnetwork genes were significantly enriched with immune-related pathways, including antigen processing and presentation, cytokine-mediated signaling pathway, modulation of synaptic transmission, and regulation of T-cell mediated cytotoxicity (Supplementary Table S14).

## 4. Discussion

The primary research goal of this study was to determine whether network-based genetic subtyping using nbPRSs derived from transcriptome signatures preserved between blood and brain tissues can identify AD subtypes that distinguish endophenotype patterns and predict future AD progression, while uncovering potential therapeutic targets for these subtypes. Three of four previously identified networks (M2, M6, and M14) were significantly associated with AD. Polygenic risk scores generated from these three networks (nbPRSs) were significantly associated with executive function and memory performance. We observed that AD incidence among CN individuals increased twofold for individuals with a high-nbPRS risk in the M14 network compared to those with a low risk in ADNI and FHS cohorts. When adjusted by *APOE* genotype or vascular risk factors, AD conversion remained relatively unchanged regarding effect size and statistical significance. Therefore, we determined that a participant’s risk status based on nbPRS is likely independent of the *APOE* genotype and several vascular risk factors. These findings indicate the potential for blood–brain M14 nbPRSs to help clinicians determine a patient’s risk for specific AD subtypes based on their transcriptomic profile and genetic risk. Patients may be classified as high AD risk before clinical symptoms appear, which will enable clinicians to perform more aggressive early screening, monitoring, and treatment strategies.

Meta-analysis of differential expression between low- and high-nbPRS subgroups for the M14 risk status identified transcriptome-wide significant genes, *HLA-DRB1* and *HLA-DQB1* (*p* < 10^−6^). Cell expression profiles of significant genes in the *HLA* locus were significantly expressed in excitatory neurons, inhibitory neurons, and oligodendrocytes, but not microglia cells; therefore, we do not believe these findings are exaggerated due to microglial enrichment. Further investigation of the M14 subnetwork revealed co-expressed genes in this subnetwork were associated with AD neuropathology and hallmark AD biomarkers, suggesting its relevance for understanding shared disease mechanisms between blood and brain tissues. Our results suggest that individuals with a high-nbPRS status for the M14 network may be at an increased risk of developing a neuroinflammatory AD subtype, with HLA types emerging as key shared markers between blood and brain tissues.

These *HLA* genes belong to the MHC class II group and encode proteins that form antigen-binding complexes on the surface of antigen-presenting immune cells, enabling the recognition of foreign peptides to initiate the adaptive immune response. The *HLA* region has been identified as one of the robust and significant loci associated with late-onset AD in GWAS studies involving participants of European ancestry [37,44]. Upregulation of MHC class II genes in activated microglia has been linked to the formation of AD-related brain lesions [45]. Additionally, single-cell transcriptome analysis has shown a positive correlation between microglial expression of *HLA-DRB1* and *HLA-DRB5* genes and AD pathology [46]. In this study, we confirmed these two genes were significantly associated with the microglial marker Iba1 level but not with the CD68 level. Since the Iba1 marker is considered a marker for all microglia [47] and CD68 is only a marker for activated microglia [48], these genes may either be associated with the process of microglial activation [44,49] or a limited number of activated microglia cells sampled in the bulk transcriptome data in our study dataset, since *HLA* genes were not significantly associated with any of the tested neuropathological markers (Figure 4B).

In addition to the *HLA* locus, other gene members of the M14 subnetwork have been previously implicated in AD pathology or related neurodegenerations. *AGER* and *ASAH1* with a predicted interaction with the Jnk pathway are linked to the development of AD or other neurological diseases, where the Jnk pathway itself being highly activated in the mammalian central nervous system (CNS) as a major regulator of key cellular processes, such as growth, proliferation, and apoptosis [50,51,52]. This regulatory pathway has been shown to correlate with both amyloid-β toxicity, through the inhibited expression of anti-apoptotic *BCL2* genes, and phosphorylated tau levels [50,53,54]. The *AGER* gene, also known as *RAGE*, has been thoroughly investigated as a critical player in the development of neurodegenerative diseases through the disruption of cellular processes, leading to neuroinflammation, neurotoxicity, and oxidative stress [55,56,57]. This gene encodes a multiligand receptor involved in plasma amyloid-beta transportation across the blood–brain barrier into the brain, and thus has been studied as a target for therapeutics [57]. A genetic variant of *ASAH1*, a ceramide-degrading lysosomal enzyme encoding gene, has been previously implicated in a GWAS study for Parkinson’s disease [58].

Additionally, *ALCAM* and *HOMER1* genes were predicted to activate the Akt signaling pathway, a pathway known to inhibit cell apoptosis and promote proliferation. *ALCAM* has been investigated in recent research as a plasma biomarker for AD, demonstrating a correlation with cognitive decline and atrophy of the medial temporal lobe [59]. Among African American individuals, *ALCAM* was one of the top loci associated with AD in an ancestry-specific GWAS, and its expression was associated with amyloid-β pathology [60]. *HOMER1* is a gene primarily expressed in the nervous system as a key mediator of synaptic plasticity and neuroinflammation, affecting postsynaptic density levels [61]. In murine models, *HOMER1* was shown to regulate amyloid-β toxicity in the early stages of AD [62].

Polygenic risk scores provide an estimate for an individual’s genetic risk for a specific disease and may have clinical use as a risk predictor by helping a clinician improve their diagnoses and preventing diseases. For Alzheimer’s disease, which is genetically complex, there is extensive research on the development of PRS to evaluate the small and large contributions of many genetic variants beyond *APOE*. In this study, we developed a blood–brain network-based PRS that predicts AD progression independent of the *APOE* genotype and vascular markers, and linked to neuroinflammatory pathways, which could be used to predict different AD subtypes.

## 5. Limitations

Study participants in all datasets (ROSMAP, MSBB, ADNI, and FHS) were predominantly non-Hispanic White, so these findings may not be indicative of individuals with other ancestries. Also, as previously described by Panitch et al., our co-expression networks were created using computational correlations to create networks rather than the underlying biological connections between genes. As an alternative, we applied IPA software to build subnetworks using biological connectedness [23]. Module eigengene correlations with AD may not fully represent the influence of technical and biological confounders. Also, eigengene values were not standardized or scaled for the correlation analysis with AD, which would improve the interpretation of effect size. There may be bias in the abundance of certain cell types in bulk brain RNA-seq data, which may influence our network structure, eigengene associations, and differential expression analysis of nbPRS subgroups. Due to the abundance of neurons in brain tissue, there may be insufficient data available for other cell types (e.g., microglia), which could explain the limited number of significant associations between *HLA* genes and microglial pathology, especially with the CD68 level. In Panitch et al.’s (2022) study, co-expression gene networks were generated using blood and brain transcriptome data from ROSMAP participants [23]. In this study, we adopted the previous Zsummary > 5 threshold, which is considered moderate rather than strong module preservation, potentially increasing the risk of identifying less stable or non-replicable network structures. However, we further evaluated these networks for the biological relevance by testing module association with AD and *APOE* ε4 status. We also used ROSMAP as a discovery dataset during our differential expression analysis to identify gene targets to profile with AD-associated quantitative traits from FHS and BUADRC patients. The use of the ROSMAP dataset in the development of blood–brain co-expression networks and in downstream analysis may influence the statistical significance of gene targets selected in differential expression analysis. Important cognitive domains (e.g., language and visuospatial domains) and AD-associated biomarkers (e.g., ptau217, ptau205, and MTBR243) were not evaluated in ADNI and ROSMAP, respectively, due to the limited availability of these traits in these cohorts. Our analysis utilized datasets from multiple cohorts, including ROSMAP, ADNI, FHS, MSBB, and BUADRC. Longitudinal cohorts, such as ADNI and FHS, provide data on diagnostic, cognitive, and neuropathological outcomes over time, which can give insights into the progression of individuals from healthy outcomes to AD during their lifetime. We also used cohorts without longitudinal data, but these cohorts had a greater sample size of AD cases and AD-related traits. Analyzing data from multiple cohorts also shows the robustness of the blood–brain nbPRSs.

## 6. Conclusions

Neuropathological heterogeneity has posed challenges for the early detection and prevention of AD. This study explored potential links between blood and brain tissues through disease-related molecular profiles associated with AD. We applied a novel approach to assess an individual’s risk of developing specific AD subtypes based on their genetic predisposition, particularly those shared across the blood and brain. Our findings demonstrate that the top-ranked network and its nbPRSs, conserved between blood and brain tissues, can distinguish biomarker patterns and predict AD progression. Notably, HLA genes emerged as key components of this network, highlighting their potential clinical utility in identifying AD subtypes associated with neuroinflammatory pathways.

## Figures and Tables

**Table 1:** Sample distribution of quartile-stratified network-based polygenic risk score (nbPRS) groups in ADNI, FHS, ROSMAP, and MSBB patients.

Dataset	Network	Low nbPRS				High nbPRS			
		N	nbPRS Mean (SD)	%Female	Age Mean (SD)	N	nbPRS Mean (SD)	%Female	Age Mean (SD)
ADNI	M2	274	52.47 (0.40)	43.43	77.91 (6.86)	274	54.63 (0.44)	39.78	77.81 (7.48)
	M6	274	25.58 (0.55)	44.89	78.34 (7.21)	274	27.82 (0.59)	41.24	76.52 (7.79)
	M14	274	69.83 (0.77)	43.80	77.52 (8.21)	274	73.40 (0.77)	42.34	77.34 (7.24)
FHS	M2	2121	58.38 (0.49)	53.70	67.59 (15.18)	2121	60.94 (0.58)	53.14	67.45 (14.53)
	M6	2121	26.95 (0.62)	54.17	67.95 (14.68)	2121	29.16 (0.52)	54.22	67.70 (14.79)
	M14	2121	141.35 (1.63)	53.65	67.76 (14.95)	2121	151.18 (2.62)	54.84	67.60 (14.65)
ROSMAP	M2	146	52.40 (0.42)	66.44	88.89 (6.44)	138	55.10 (1.34)	64.49	88.36 (7.05)
	M6	140	25.50 (0.54)	70.00	88.64 (7.13)	144	27.70 (0.59)	64.58	88.88 (6.35)
	M14	155	69.50 (0.66)	60.00	89.01 (6.55)	141	73.40 (1.09)	60.99	88.49 (6.48)
MSBB	M2	49	48.70 (0.59)	34.69	82.59 (7.81)	53	52.50 (1.58)	35.85	81.15 (7.72)
	M6	56	26.70 (0.46)	28.57	83.91 (6.75)	55	29.10 (0.60)	43.64	81.93 (7.72)
	M14	49	77.40 (1.33)	34.69	82.94 (7.73)	55	84.30 (1.37)	43.64	80.38 (7.67)

ADNI: Alzheimer’s Disease Neuroimaging Initiative; FHS: Framingham Heart Study; ROSMAP: Religious Orders Study and Rush Memory and Aging Project; MSBB: Mount Sinai Brain Bank; nbPRS: network-based polygenic risk score; low nbPRS = 1st quartile; high nbPRS = 4th quartile; %Female: percentage of female participants; Age: age at exam for ADNI and FHS participants and age at death for ROSMAP and MSBB brains; SD: standard deviation.

**Table 2:** Cox proportional hazards ratio for conversion to AD in ADNI and FHS patients comparing network-based PRS groups by network.

Network	ADNI		FHS	
	HR (95% CI)	*p*-Value	HR (95% CI)	*p*-Value
M2	2.01 (1.15–3.52)	0.014	1.64 (1.29–2.08)	5.3 × 10^−5^
M6	1.51 (0.88–2.59)	0.130	1.49 (1.15–1.94)	0.029
M14	2.01 (1.13–3.56)	0.018	2.11 (1.63–2.74)	1.9 × 10^−8^

ADNI: Alzheimer’s Disease Neuroimaging Initiative; FHS: Framingham Heart Study. Cox proportional hazards ratio (HR) and 95% confidence interval (CI) were computed between high-risk and low-risk groups based on network-based polygenic risk scores (nbPRSs).

**Table 3:** Differentially expressed genes with *p* < 0.001 in the meta-analysis of ROSMAP and MSBB datasets.

Network	Gene Name	ROSMAP		MSBB BM10		Meta-Analysis	
		logFC	*p*-Value	logFC	*p*-Value	Z-Score	*p*-Value
M2	*ZNF483*	−0.15	8.40 × 10^−4^	−0.17	0.0179	−4.07	4.78 × 10^−5^
M14	***HLA-DRB1***	**0.57**	**1.30 × 10^−5^**	**0.55**	**3.62 × 10^−3^**	**5.22**	**1.77 × 10^−7^**
	***HLA-DQB1***	**0.92**	**2.19 × 10^−5^**	**0.76**	**3.24 × 10^−3^**	**5.14**	**2.79 × 10^−7^**
	*HLA-DRB5*	1.14	3.78 × 10^−5^	0.58	0.0375	4.61	3.97 × 10^−6^
	*SOHLH1*	−0.19	1.14 × 10^−3^	−0.20	0.0451	−3.82	1.35 × 10^−4^
	*PLXNA1*	−0.10	2.07 × 10^−3^	−0.15	0.0490	−3.65	2.65 × 10^−4^
	*WDR44*	0.09	8.10 × 10^−3^	0.09	8.27 × 10^−3^	3.59	3.28 × 10^−4^
	*MCM3*	0.08	5.83 × 10^−3^	0.12	0.0229	3.51	4.46 × 10^−4^
	*BTD*	0.09	1.81 × 10^−2^	0.14	2.95 × 10^−3^	3.50	4.62 × 10^−4^
	*NEUROD2*	−0.13	8.38 × 10^−3^	−0.22	0.0168	−3.46	5.38 × 10^−4^
	*SGCD*	0.09	1.46 × 10^−2^	0.15	6.63 × 10^−3^	3.45	5.66 × 10^−4^
	*MMP17*	−0.14	9.16 × 10^−3^	−0.23	1.62 × 10^−3^	−3.44	5.79 × 10^−4^
	*GABARAPL2*	0.07	8.90 × 10^−3^	0.12	0.0250	3.37	7.48 × 10^−4^
	*TRPM3*	0.11	2.57 × 10^−2^	0.19	3.57 × 10^−3^	3.36	7.86 × 10^−4^
	*PKDCC*	−0.10	7.96 × 10^−3^	−0.13	0.0325	−3.36	7.94 × 10^−4^
	*PLK1*	−0.10	1.61 × 10^−2^	−0.27	0.0127	−3.31	9.35 × 10^−4^

ROSMAP: Religious Orders Study and Rush Memory and Aging Project; MSBB BM10: Brodmann area 10 (BM10) of the prefrontal cortex area in Mount Sinai Brain Bank (MSBB). Log2 fold change (logFC) and *p* value (*p*-value) from differential expression between AD and control brains was obtained in the meta-analysis of ROSMAP and MSBB. Transcriptome-wide significance level with *p* < 10^−6^) indicated in bold.

## Data Availability

Genotypes and clinical/neuropathological phenotype data are accessible by directly applying to the LONI portal for the ADNI at http://adni.loni.usc.edu. Summary statistics for GWAS can be accessed by applying directly to the National Institute on Aging Genetics of Alzheimer’s Disease Data Storage Site (NIAGADS), a NIA/NIH-sanctioned qualified-access data repository, under accession NG00075. Data supporting the findings of this study are available from the NIAGADS website (https://www.niagads.org/).

## Supplementary Materials

The following supporting information can be downloaded at: https://www.mdpi.com/article/10.3390/jcm15082885/s1. Supplementary Table S1: Sample size of longitudinal data stratified by cognitive AD diagnosis in ADNI1-2/GO and FHS; Supplementary Table S2: Sample size stratified by neuropathological AD diagnosis in ROSMAP and MSBB; Supplementary Table S3: AD related co-expressed gene networks in ROSMAP; Supplementary Table S4: Distribution of nbPRS in ADNI, FHS, ROSMAP, and MSBB; Supplementary Table S5: Cox proportional hazard ratio for nbPRS subgroups stratified by median, quartile, and tertile in FHS; Supplementary Table S6: Sample size of nbPRS risk stratification by tertile in FHS; Supplementary Table S7: *APOE* ε4-stratified Cox proportional hazard ratio for nbPRS subgroups in FHS; Supplementary Table S8: Cox proportional hazard ratio adjusted by APOE genotype and vascular risk factors for nbPRS subgroups in FHS; Supplementary Table S9: Differential expression analysis of genes for M14 nbPRS in ROSMAP; Supplementary Table S10: Differential expression analysis of genes in subnetwork 1 for M14 nbPRS in meta-analyzed ROSMAP and MSBB BM10, and AD status in FHS; Supplementary Table S11: Profile of M14 subnetwork 1 genes with tau-related quantitative outcomes; Supplementary Table S12: Profile of M14 subnetwork 1 genes with amyloid-related quantitative outcomes; Supplementary Table S13: Profile of M14 subnetwork 1 genes with neuroinflammatory-related quantitative outcomes; Supplementary Table S14: Significant pathways of IPA subnetwork 1 genes; Supplementary Figure S1: Distributions of nbPRS for module M2, M6, and M14 in ROSMAP, MSBB, ADNI, and FHS; Supplementary Figure S2: APOE ε4 stratified survival curves for nbPRS subgroups in FHS using Cox proportional hazards model; Supplementary Figure S3: Survival curves for nbPRS subgroups in FHS using Cox proportional hazards model adjusted by APOE genotype and several vascular risk factors; Supplementary Figure S4: Cell expression profile of top differentially expressed genes (DEGs) in ROSMAP; Supplementary Figure S5: Linkage disequilibrium (LD) structure of the *HLA* locus.
