# Supplementary material for: Blood–Brain Network-Based Polygenic Risk Scores Reveal Biomarker Signatures and the Progression of Alzheimer’s Disease
Source: J Clin Med. 2026 Apr 10;15(8):2885. doi: 10.3390/jcm15082885 (PMC13116307; doi:10.3390/jcm15082885)

SUPPLEMENTARY INFORMATION

Supplementary Table S1: Sample size of longitudinal data stratified by cognitive AD diagnosis in ADNI1-2/GO and FHS.

Characteristics	ADNI 1-2/GO			FHS		
	CN	MCI	AD	CN	MCI	AD
Baseline (N)	315	610	197	8,310	0	0
Last Exam (N)	284	369	456	7,460	310	540
Mean Age at Baseline (SD)	75.29 (5.29)	73.41 (7.52)	75.38 (8.05)	39.49 (7.10)	39.45 (7.47)	41.29 (7.63)
Mean Age at Last (SD)	79.50 (6.99)	78.51 (7.82)	78.58 (7.68)	81.05 (10.65)	77.38 (11.25)	84.37 (7.56)
ε4+ (%)	23.9	39.6	65.1	20.5	24.5	31.5
Female (%)	46.5	39.8	41.9	53.2	54.2	67.2

ADNI1-2/GO: Alzheimer's Disease Neuroimaging Initiative cohorts 1 and 2/GO; FHS: Framingham Heart Study; Age at Baseline: age at first exam, expressed as mean (standard deviation, SD); Age at Last: age at last exam, expressed as mean (standard deviation, SD); AD: Alzheimer's Disease; CN: cognitively normal; MCI: mild cognitive impairment; N: number of subjects; ε4+: subjects with APOE ε4 genotype.

Supplementary Table S2: Sample size stratified by neuropathological AD diagnosis in ROSMAP and MSBB.

Path DX	ROSMAP				MSBB			
	N	ε4+ (%)	Female (%)	Age at Death Mean (SD)	N	ε4+ (%)	Female (%)	Age at Death Mean (SD)
AD	775	33.6	29.9	90.3(6.4)	71	7.04	56.3	80.0(8.6)
Ctrl	440	13.4	41.1	87.3(7.1)	252	25.4	80.1	83.9(7.3)

ROSMAP: Religious Orders Study and the Rush Memory and Aging Project; MSBB: Mount Sinai Brain Bank; Path DX: pathological diagnosis; AD: autopsy-confirmed Alzheimer's Disease diagnosis; Ctrl: not AD diagnosis; N: number of subjects; ε4+: percentage of subjects with APOE ε4 genotype. APOE. Age at Death is expressed as mean (standard deviation).

Supplementary Table S3: AD related co-expressed gene networks in ROSMAP.

Network	No. Genes	$Z_{summary}$	AD vs. Control			*APOE* ε4+ vs. ε4-		
			**Corr**	**P-value**	**P-adj**	**Corr**	**P-value**	**P-adj**
M2	2297	8.00	-0.09	0.03	0.04	0.01	0.80	0.9
M6	1044	6.10	-0.10	0.01	0.02	0.01	0.80	0.9
M7	230	5.77	0.06	0.10	0.10	0.11	4.0×10^{-3}	0.02
M14	4169	5.08	-0.11	6.0×10^{-3}	0.02	-0.01	0.90	0.9

Co-expression networks selected based on correlation with AD diagnosis or APOE ε4 genotype and preservation in ROSMAP blood and brain with a $Z_{summary}$ score > 5. No. Genes: number of gene members varied among networks; Corr: correlation coefficient; P-value; correlation p-value; P-adj: adjusted p-value using Benjamini Hochberg correction. We assessed correlation between networks and either AD diagnosis or APOE ε4 genotype.

Supplementary Table S4: Distribution of nbPRS in ADNI, FHS, ROSMAP, and MSBB.

Network	ADNI<br>**Mean PRS (SD)**	FHS<br>**Mean PRS (SD)**	ROSMAP<br>**Mean PRS (SD)**	MSBB<br>**Mean PRS (SD)**
M2	53.53 (0.85)	59.65 (1.01)	53.62 (1.22)	50.38 (1.64)
M6	26.67 (0.92)	28.05 (0.91)	26.58 (0.90)	27.88 (0.97)
M14	71.60 (1.42)	146.01 (3.94)	71.38 (1.55)	80.68 (2.75)

Mean and standard deviation of the network-based polygenic risk scores (nbPRS) for preserved and AD significant networks. PRS were calculated for each network gene set (nbPRS) using SNPs within the gene region (±20k bases). SNPS were collected from AD GWAS summary statistics (Kunkle *et al.*, 2019) with a p-value < 10^{-3} and filtered using minor allele frequency (<1%), imputation quality (R^2<0.4), and linkage disequilibrium (R^2>0.5).

Supplementary Table S5: Cox proportional hazard ratio for nbPRS subgroups stratified by median, quartile, and tertile in FHS.

Network	Median		Quartile		Tertile	
	HR (95% CI)	P-value	HR (95% CI)	P-value	HR (95% CI)	P-value
M2	1.42 (1.20 – 1.69)	4.53e-05	1.64 (1.29 - 2.08)	5.34e-05	1.56 (1.09 – 2.23)	1.54e-02
M6	1.22 (0.95 – 1.42)	1.85e-02	1.49 (1.15 - 1.94)	2.86e-02	2.30 (1.53 – 3.53)	1.26e-04
M14	1.60 (1.33 – 1.91)	4.06e-07	2.11 (1.63 - 2.74)	1.89e-08	1.17 (0.78 – 1.75)	1.19e-08

Cox Proportional Hazard Ratio with 95% confidence interval computed for high-risk subgroup survival using the low-risk subgroup as a reference. Median: risk stratification by median, low nbPRS < median and high nbPRS > median; Quartile: risk stratification by quartiles, low nbPRS = 1st quartile and high nbPRS = 4th quartile; Tertile: risk stratification by tertile, low nbPRS = 1st tertile and high nbPRS = 10th tertile; HR: Cox Proportional Hazard Ratio; CI: Confidence Interval.

Supplementary Table S6: Sample size of nbPRS risk stratification by tertile in FHS.

Tertile	Total (N)	AD (N)	ε4+ (%)	Female (%)	Age at Last Mean(SD)
1	849	53	19.32%	43.70%	67.30 (14.9)
2	848	37	20.99%	46.82%	67.90 (14.9)
3	848	45	17.10%	48.11%	67.50 (15.6)
4	848	38	21.70%	43.87%	68.00 (14.8)
5	848	56	20.17%	44.22%	67.80 (15.2)
6	848	44	20.64%	45.75%	67.00 (15.4)
7	848	61	19.58%	45.40%	66.80 (14.5)
8	848	74	23.47%	47.88%	67.70 (14.9)
9	848	53	23.94%	47.52%	67.60 (14.2)
10	848	79	26.42%	45.99%	67.50 (14.6)

Cox Proportional Hazard Ratio with 95% confidence interval computed for high-risk subgroup survival using the low-risk subgroup as a reference. Median: risk stratification by median, low nbPRS < median and high nbPRS > median; Quartile: risk stratification by quartiles, low nbPRS = 1st quartile and high nbPRS = 4th quartile; Tertile: risk stratification by tertile, low nbPRS = 1st tertile and high nbPRS = 10th tertile; HR: Cox Proportional Hazard Ratio; CI: Confidence Interval.

Supplementary Table S7: *APOE* ε4-stratified Cox proportional hazard ratio for nbPRS subgroups in FHS.

**Network**	**ε4-**		**ε4+**	
	**HR (95% CI)**	**P-value**	**HR (95% CI)**	**P-value**
M2	1.64 (1.22 - 2.21)	1.01e-03	1.28 (0.82 - 2.01)	2.78e-01
M6	1.48 (1.06 – 2.06)	2.14e-02	1.02 (0.64 - 1.61)	9.37e-01
M14	1.63 (1.18 - 2.26)	3.38e-03	1.53 (0.74 - 3.15)	2.49e-01

Cox Proportional Hazard Ratio with 95% confidence interval computed for high nbPRS subgroup survival using the low nbPRS subgroup as a reference. HR: Cox Proportional Hazard Ratio; CI: Confidence Interval..

Supplementary Table S8: Cox proportional hazard ratio adjusted by APOE genotype and vascular risk factors for nbPRS subgroups in FHS.

**Network**	***+APOE***		**+LDL+Glucose**	
	**HR (95% CI)**	**P-value**	**HR (95% CI)**	**P-value**
M2	1.64 (1.28 - 2.09)	7.56e-05	1.42 (1.01 - 2.10)	4.20e-02
M6	1.39 (1.06 – 1.82)	1.68e-02	1.57 (1.04 - 2.38)	3.26e-02
M14	1.90 (1.45 - 2.49)	3.76e-06	2.11 (1.44 - 3.10)	1.36e-04

Cox Proportional Hazard Ratio with 95% confidence interval computed for high nbPRS subgroup survival using the low nbPRS subgroup as a reference. HR: Cox Proportional Hazard Ratio; LDL: Low-density Lipoprotein; Glucose: Fasting Blood Glucose.

Supplementary Table S9: Differential expression analysis of genes for M14 nbPRS in ROSMAP.

Gene Name	nbPRS High vs. Low	
	LogFC	P-value
HLA-DRB1	0.565	1.30E-05
HLA-DQB1	0.917	2.19E-05
NUTM2B	-0.319	3.76E-05
HLA-DRB5	1.140	3.78E-05
C10orf142	-0.259	1.66E-04
HLA-DQA2	-0.901	1.75E-04
HPS4	0.071	2.64E-04
KBTBD4	0.168	4.53E-04
TRMT1	-0.091	6.16E-04
FAM153B	-0.204	7.59E-04
DHX8	0.078	8.03E-04
HHLA3	-0.143	8.36E-04
TTC4	-0.079	8.46E-04
MFSD13A	-0.107	9.91E-04

nbPRS: network-based polygenic risk score; LogFC: Log_{10} fold change; Low: nbPRS < 1st Quartile; High: nbPRS > 3rd Quartile.

Supplementary Table S10: Differential expression analysis of genes in subnetwork 1 for M14 nbPRS in meta-analyzed ROSMAP and MSBB BM10, and AD status in FHS.

Gene Name	nbPRS High vs. Low		AD vs. Control	
	**Z-score**	**P-value**	**Z-score**	**P-value**
HLA-DRB1	5.22	1.77E-07	0.99	0.32
HLA-DQB1	5.14	2.79E-07	1.46	0.15
HLA-DRB5	4.61	3.97E-06	0.75	0.45
SDF4	-3.19	1.43E-03	0.08	0.94
PUS1	-3.13	1.77E-03	-0.83	0.41
NBR1	3.01	2.63E-03	0.56	0.58
HLA-C	2.99	2.79E-03	1.43	0.15
EIF4B	2.99	2.84E-03	-0.26	0.79
GRM3	2.98	2.90E-03	-1.32	0.19
MMP25	-2.95	3.16E-03	1	0.32
UBE2I	-2.94	3.26E-03	-0.38	0.7
TNFRSF17	-2.90	3.77E-03	-0.16	0.87
AGER	-2.80	5.15E-03	-0.63	0.53
TNFRSF25	-2.76	5.78E-03	1.47	0.14
ALCAM	2.71	6.78E-03	0.49	0.62
ASAH1	2.70	7.00E-03	-3.17	1.50E-03
NDUFB9	2.70	7.05E-03	-0.47	0.64
EXOC3	2.63	8.44E-03	-2.51	0.012
HOMER1	-2.63	8.53E-03	-1.31	0.19
GP6	-2.62	8.87E-03	-0.23	0.82
DUSP16	2.60	9.24E-03	3.69	2.20E-04
ENO1	2.60	9.25E-03	1.57	0.12
RPN2	2.58	9.79E-03	0.19	0.85

Genes are listed in order of p-value significance for nbPRS high vs. low.

Supplementary Table S11: Profile of M14 subnetwork 1 genes with tau-related quantitative outcomes.

Gene Name	Braak		AT8		pTau181		pTau202		pTau231		pTau396	
	Beta	P-value	Beta	P-value	Beta	P-value	Beta	P-value	Beta	P-value	Beta	P-value
AGER	-0.07	0.57	0.19	0.13	0.32	0.02	0.28	0.03	0.35	8.78E-03	0.28	0.05
ALCAM	0.06	0.64	0.19	0.12	0.35	3.59E-03	0.17	0.18	0.53	8.93E-06	0.17	0.21
ASAH1	-0.30	0.07	-0.05	0.76	0.22	0.18	0.04	0.80	0.44	8.36E-03	-0.26	0.14
DUSP16	0.11	0.46	0.40	6.42E-03	0.40	7.15E-03	0.25	0.09	0.56	1.54E-04	0.32	0.05
EIF4B	-0.35	0.08	-0.05	0.80	0.66	1.29E-03	-0.04	0.83	0.66	9.58E-04	-0.15	0.51
ENO1	-0.07	0.62	0.23	0.08	0.40	2.06E-03	0.29	0.32	0.53	7.73E-05	-0.01	0.93
EXOC3	-0.16	0.23	0.04	0.73	0.58	5.32E-06	0.11	0.43	0.49	1.70E-04	3.47E-03	0.98
GP6	-0.14	0.20	0.11	0.31	0.36	1.36E-03	-0.07	0.54	0.29	6.04E-03	-0.12	0.30
GRM3	-0.17	0.28	0.19	0.21	0.61	2.29E-05	0.08	0.59	0.62	4.37E-05	0.13	0.43
HLA-C	-0.04	0.76	-0.15	0.19	-0.02	0.90	0.19	0.12	-0.07	0.58	-0.04	0.74
HLA-DQB1	-0.01	0.90	-0.02	0.69	-0.06	0.31	0.05	0.33	-0.01	0.90	-0.02	0.78
HLA-DRB1	-0.02	0.83	0.01	0.87	0.05	0.52	0.19	0.01	0.10	0.15	0.02	0.81
HLA-DRB5	-2.06E-03	0.96	-0.04	0.31	-0.04	0.42	0.09	0.06	-0.01	0.83	0.01	0.80
HOMER1	-0.10	0.27	0.04	0.62	0.09	0.35	0.02	0.85	0.17	0.07	-0.02	0.87
MMP25	0.01	0.91	0.14	0.17	0.33	5.04E-03	0.14	0.19	0.28	8.22E-03	0.01	0.95
NBR1	-0.28	0.19	0.24	0.27	0.70	4.71E-04	0.08	0.67	0.87	2.39E-05	0.08	0.72
NDUFB9	-0.12	0.47	-0.01	0.94	0.28	0.09	0.02	0.90	0.33	0.05	0.01	0.97
PUS1	-0.20	0.08	0.09	0.43	0.36	1.40E-03	0.04	0.71	0.40	3.79E-04	0.01	0.94
RPN2	-0.14	0.04	0.17	0.29	0.38	0.01	0.31	0.60	0.68	2.09E-05	-0.03	0.85
SDF4	-0.22	0.18	0.22	0.17	0.62	7.28E-05	0.18	0.26	0.59	2.13E-04	0.01	0.95
TNFRSF17	-0.10	0.27	-0.15	0.08	0.02	0.81	0.25	9.61E-03	0.03	0.77	0.16	0.13
TNFRSF25	-0.12	0.15	0.09	0.24	0.20	0.02	0.02	0.77	0.18	0.02	-0.06	0.47
UBE2I	-0.23	0.26	0.26	0.21	0.68	5.91E-04	0.18	0.38	0.68	8.07E-04	-0.11	0.61

Braak: Braak score; AT8: anti-tau monoclonal antibody 8; pTau181: phosphorylated tau at 181; pTau202: phosphorylated tau at 202; pTau231: phosphorylated tau at 231; pTau396: phosphorylated tau at 396. Genes are listed in alphabetical order.

Supplementary Table S12: Profile of M14 subnetwork 1 genes with amyloid-related quantitative outcomes.

Gene Name	Cerad		4G8		Ab40		Ab42	
	**Beta**	**P-value**	**Beta**	**P-value**	**Beta**	**P-value**	**Beta**	**P-value**
AGER	-0.10	0.48	2.28E-03	0.99	-0.11	0.41	0.09	0.52
ALCAM	0.03	0.81	0.16	0.22	-2.32E-03	0.99	0.10	0.42
ASAH1	-0.28	0.10	-1.21E-03	0.99	-0.22	0.19	0.03	0.86
DUSP16	0.12	0.46	0.14	0.38	-0.14	0.36	0.06	0.71
EIF4B	-0.17	0.41	-0.02	0.91	-0.11	0.61	0.03	0.88
ENO1	-0.05	0.70	0.08	0.56	0.07	0.63	0.13	0.36
EXOC3	-0.03	0.82	0.01	0.95	0.08	0.56	0.06	0.65
GP6	-0.02	0.84	0.17	0.13	0.05	0.64	3.36E-03	0.98
GRM3	-0.18	0.26	0.17	0.29	-0.08	0.61	0.04	0.79
HLA-C	-0.16	0.21	-0.10	0.43	-0.14	0.27	0.03	0.82
HLA-DQB1	1.32E-03	0.98	0.09	0.10	3.81E-03	0.94	0.05	0.37
HLA-DRB1	-0.05	0.50	0.07	0.34	-0.07	0.31	-0.03	0.74
HLA-DRB5	-0.03	0.57	2.08E-03	0.97	0.01	0.79	-0.02	0.74
HOMER1	-0.06	0.52	0.10	0.32	0.05	0.58	0.10	0.28
MMP25	-0.14	0.19	0.09	0.45	-0.04	0.74	-0.08	0.49
NBR1	-0.04	0.85	0.13	0.56	-0.17	0.44	0.10	0.64
NDUFB9	-0.23	0.17	-0.15	0.38	-0.03	0.86	-0.06	0.72
PUS1	-0.06	0.63	0.02	0.84	0.02	0.83	0.11	0.35
RPN2	-0.16	0.35	0.11	0.53	-0.12	0.48	0.09	0.58
SDF4	-0.10	0.57	-0.03	0.88	4.59E-03	0.98	0.10	0.56
TNFRSF17	-0.17	0.07	-0.23	0.02	-0.21	0.03	-0.15	0.12
TNFRSF25	-0.02	0.83	0.07	0.41	0.02	0.80	0.05	0.56
UBE2I	-0.05	0.81	4.66E-03	0.98	0.12	0.58	0.22	0.29

Cerad: Cerad score; 4G8: anti-amyloid monoclonal antibody 4G8; Ab40; amyloid-beta protein 40; Ab42: amyloid-beta protein 42. Genes are listed in alphabetical order.

Supplementary Table S13: Profile of M14 subnetwork 1 genes with neuroinflammatory-related quantitative outcomes.

Gene Name	Iba1		CD68		C4A		C4B		C1q	
	**Beta**	**P-value**	**Beta**	**P-value**	**Beta**	**P-value**	**Beta**	**P-value**	**Beta**	**P-value**
AGER	0.50	1.89E-04	-0.05	0.71	0.29	0.028	0.06	0.66	4.52E-05	1.00
ALCAM	0.55	7.29E-06	0.02	0.87	0.37	2.10E-03	0.07	0.59	0.13	0.28
ASAH1	0.78	3.16E-06	0.14	0.43	0.57	5.55E-04	-0.25	0.14	0.20	0.23
DUSP16	0.49	1.51E-03	0.21	0.17	0.11	0.47	-0.08	0.62	-0.01	0.96
EIF4B	0.77	1.74E-04	-0.10	0.65	0.60	3.52E-03	-0.35	0.11	0.06	0.75
ENO1	0.53	8.80E-05	0.13	0.34	0.51	7.28E-05	0.05	0.71	0.15	0.25
EXOC3	0.50	1.83E-04	0.01	0.96	0.58	5.84E-06	-0.04	0.78	0.14	0.30
GP6	0.26	0.02	0.07	0.55	0.43	5.18E-05	-2.06E-04	1.00	0.15	0.16
GRM3	0.36	0.02	-0.14	0.38	0.55	1.83E-04	-0.09	0.57	0.22	0.15
HLA-C	0.35	4.62E-03	0.11	0.38	-0.06	0.63	-0.05	0.68	0.22	0.07
HLA-DQB1	0.09	0.09	0.03	0.61	0.04	0.45	0.05	0.37	0.03	0.54
HLA-DRB1	0.35	1.11E-06	0.08	0.27	2.19E-03	0.98	0.04	0.59	0.11	0.11
HLA-DRB5	0.14	1.50E-03	0.06	0.22	0.02	0.59	0.07	0.16	0.08	0.08
HOMER1	0.25	9.31E-03	-0.01	0.95	0.21	0.02	-0.02	0.82	0.15	0.10
MMP25	0.42	9.83E-05	0.06	0.59	0.22	0.04	0.10	0.36	0.16	0.13
NBR1	0.76	4.25E-04	-0.13	0.54	0.70	5.58E-04	-0.19	0.37	0.21	0.32
NDUFB9	0.50	2.96E-03	-0.03	0.86	0.56	4.61E-04	-0.14	0.40	0.04	0.80
PUS1	0.40	4.37E-04	0.03	0.77	0.38	6.36E-04	-0.10	0.40	0.08	0.48
RPN2	0.79	9.41E-07	0.19	0.25	0.49	1.70E-03	-0.04	0.80	0.17	0.28
SDF4	0.65	6.63E-05	0.04	0.82	0.63	5.76E-05	-0.02	0.92	0.17	0.30
TNFRSF17	0.16	0.09	-0.12	0.21	0.13	0.15	0.04	0.69	0.04	0.66
TNFRSF25	0.21	0.01	-0.01	0.90	0.21	7.49E-03	0.03	0.73	0.07	0.40
UBE2I	0.73	3.81E-04	0.15	0.49	0.71	3.97E-04	-0.20	0.36	0.25	0.22

Iba1: ionized calcium binding adaptor molecule 1; CD68: cluster of differentiation 68; C4A: complement component 4A; C4B: complement component 4B; C1q: complement component 1. Genes are listed in alphabetical order.

Supplementary Table S14: Significant pathways of IPA subnetwork 1 genes.

Pathway	Combined Score	OR	P-value	P-adj	Genes
Antigen processing and presentation	398.5	34.7	1.02E-05	9.33E-05	HLA-DRB5;HLA-C;HLA-DRB1;HLA-DQB1
interferon-gamma-mediated signaling pathway (GO:0060333)	483.0	40.1	5.91E-06	0.002	HLA-DRB5;HLA-C;HLA-DRB1;HLA-DQB1
antigen processing and presentation of peptide antigen via MHC class II (GO:0002495)	280.6	26.7	2.73E-05	0.004	HLA-DRB5;MARCHF8;HLA-DRB1;HLA-DQB1
cellular response to interferon-gamma (GO:0071346)	213.6	21.9	5.77E-05	0.006	HLA-DRB5;HLA-C;HLA-DRB1;HLA-DQB1
regulation of CD4-positive, alpha-beta T cell activation (GO:2000514)	1895.0	201.6	8.28E-05	0.007	AGER;HLA-DRB1
MHC class II receptor activity (GO:0032395)	1349.73	151.19	1.33E-04	7.56E-03	HLA-DRB1;HLA-DQB1
G protein-coupled glutamate receptor signaling pathway (GO:0007216)	752.1	93.0	3.08E-04	0.020	GRM3;HOMER1
cytokine-mediated signaling pathway (GO:0019221)	47.7	6.5	6.59E-04	0.031	HLA-DRB5;TNFRSF17;HLA-C;TNFRSF25;HLA-DRB1;HLA-DQB1
antigen processing and presentation of exogenous peptide antigen via MHC class II (GO:0019886)	143.4	19.6	6.66E-04	0.031	HLA-DRB5;HLA-DRB1;HLA-DQB1
antigen processing and presentation of exogenous peptide antigen (GO:0002478)	133.5	18.6	7.70E-04	0.031	HLA-DRB5;HLA-DRB1;HLA-DQB1
modulation of chemical synaptic transmission (GO:0050804)	123.0	17.6	9.08E-04	0.032	GRM3;HOMER1;AGER
mitogen-activated protein kinase binding (GO:0051019)	302.03	44.75	1.17E-03	0.033	NBR1;DUSP16
regulation of T cell mediated cytotoxicity (GO:0001914)	302.0	44.8	1.17E-03	0.038	AGER;HLA-DRB1
single-stranded RNA binding (GO:0003727)	164.96	28.08	2.81E-03	0.053	PUS1;EIF4B

OR: odds ratio; Padj: adjusted p-value using Benjamini-Hochberg correction for multiple testing; Combined Score: enrichment score computed by enrichR.

Supplementary Figure S1: Distributions of nbPRS for module M2, M6, and M14 in ROSMAP, MSBB, ADNI, and FHS.

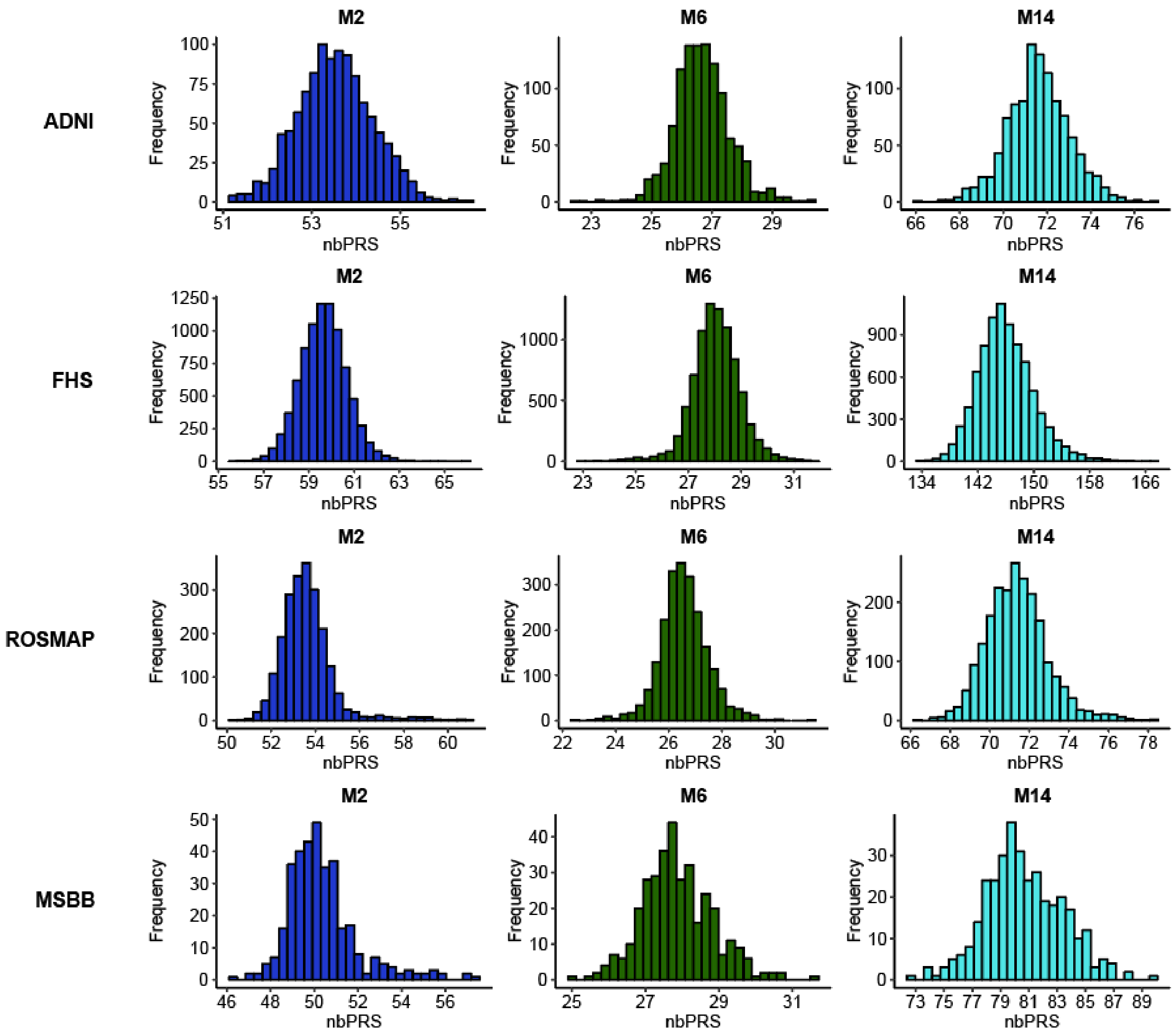

Supplementary Figure S2: APOE ε4 stratified survival curves for nbPRS subgroups in FHS using Cox proportional hazards model. **A.** Survival curves for APOE ε4- subjects. **B.** Survival curves for subjects with at least one copy of the APOE e4 risk allele. The reference group contains subjects with undetermined nbPRS risk. All models conduct left side censoring using baseline age less than 75 and age at last greater than 65.

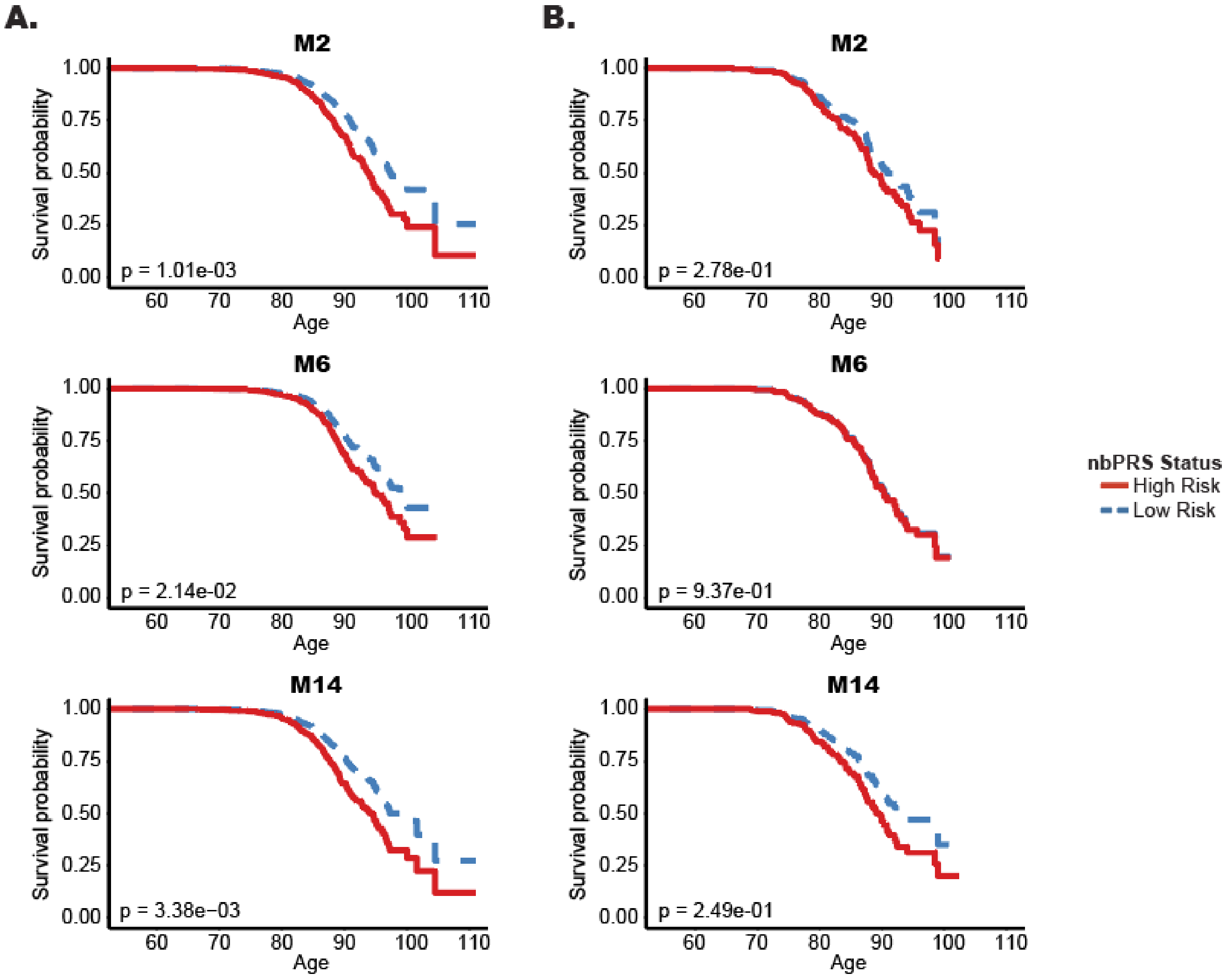

Supplementary Figure S3: Survival curves for nbPRS subgroups in FHS using Cox proportional hazards model adjusted by APOE genotype and several vascular risk factors. Cox proportional hazards models were adjusted by sex, age, family structure, and either (A) APOE genotype or (B) low-density lipoprotein (LDL) and fasting blood glucose (FBG) as vascular risk factors. Low-nbPRS subgroup is represented by the dotted blue curve, and high-nbPRS by the solid red curve. All models conduct left side censoring using baseline age less than 75 and age at last greater than 65.

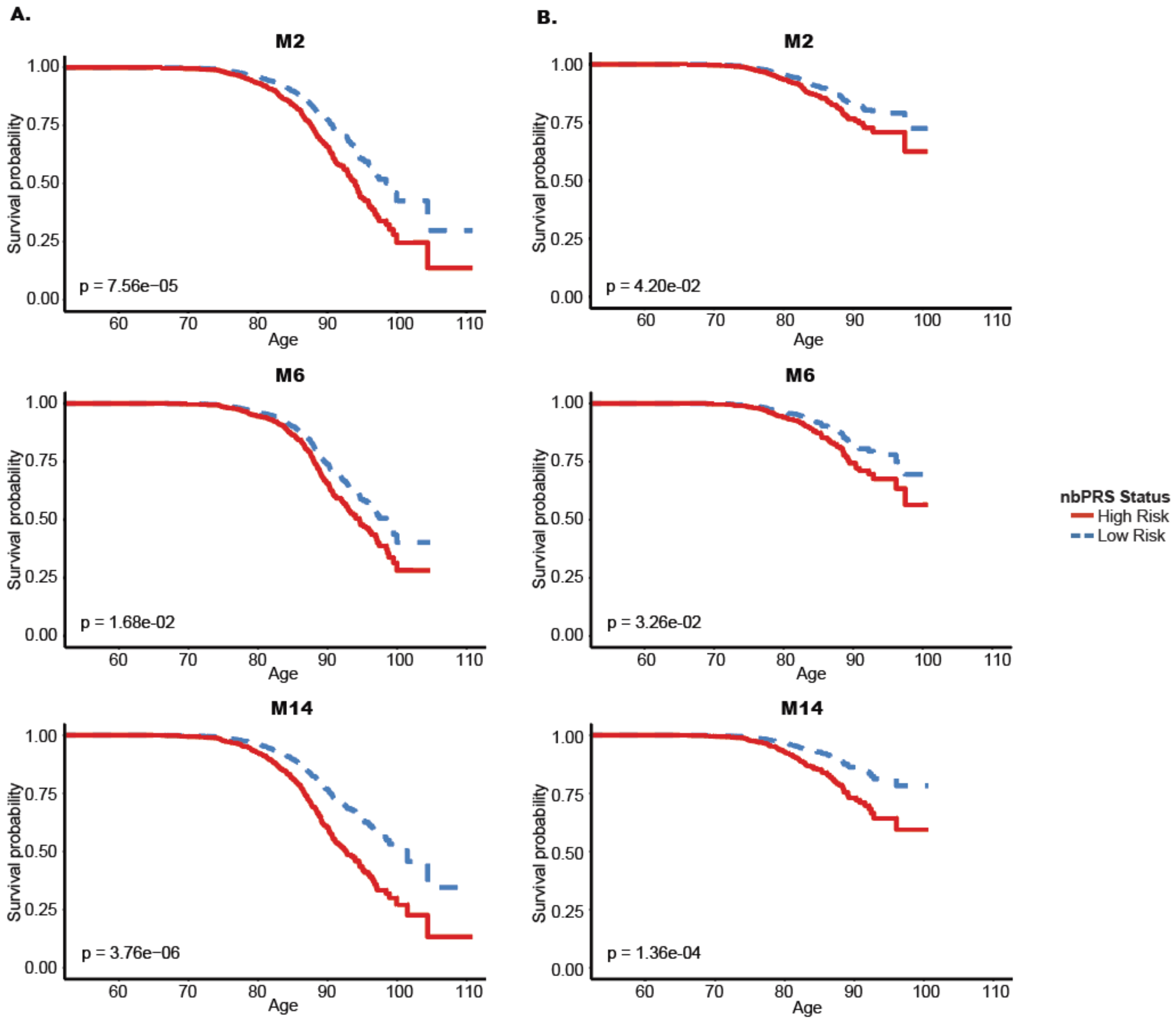

Supplementary Figure S4: Cell expression profile of top differentially expressed genes (DEGs) in ROSMAP. Cell-specific expression levels of significant DEGs in high- vs. low-nbPRS subgroups (P < 0.001) were previously measured as reported in *Panitch et. al 2022*[23]. AST: Astrocyte; END: Endothelial Cell; EXN: Excitatory Neuron; INN: Inhibitory Neuron; MIC: Microglia; OLI: Oligodendrocyte; OPC: Oligodendrocyte Precursor Cell; logFC: Log Fold Change.

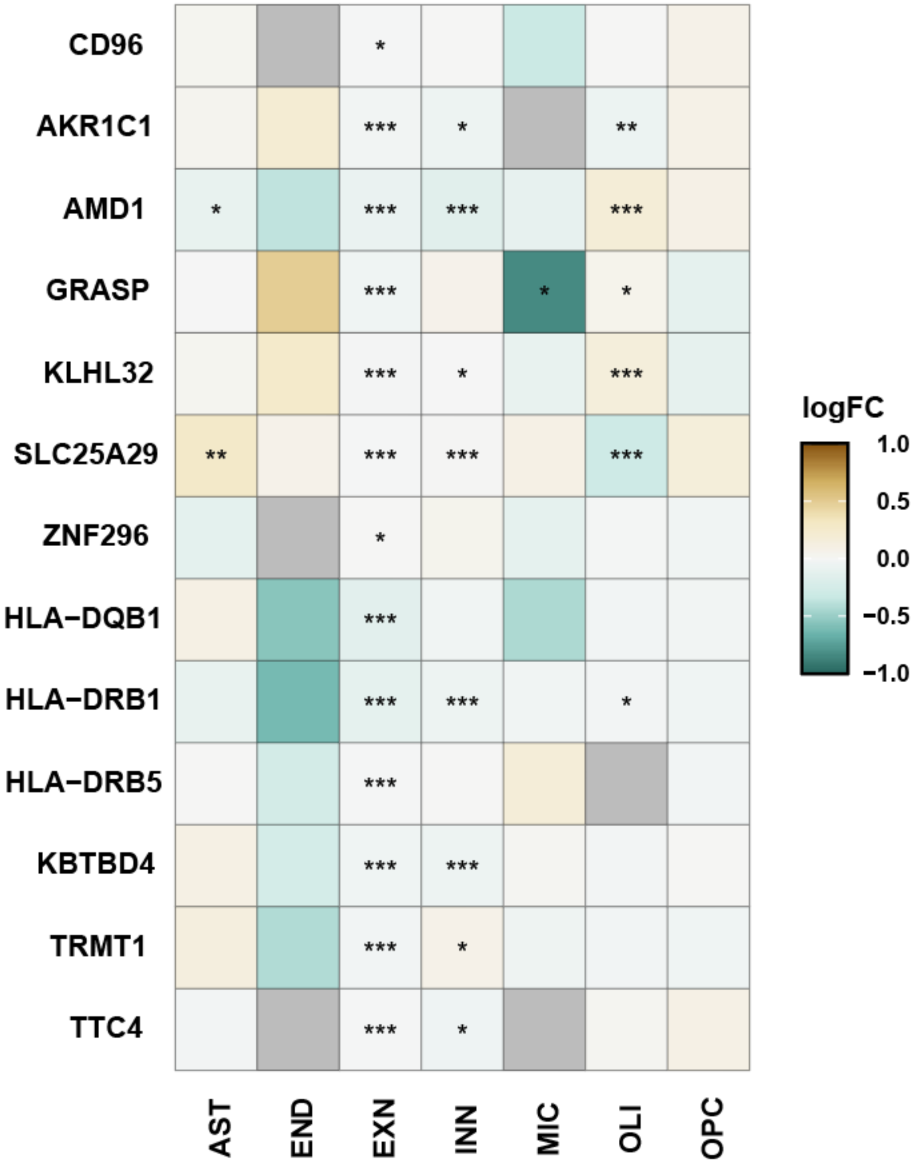

Supplementary Figure S5: Linkage disequilibrium (LD) structure of the *HLA* locus. SNPs with $P<10^{-6}$ on chromosome 6 were selected from the nbPRS for the M14 network. LD was calculated using r^2 based (red color) and D' based (blue color) approach using the 1000 Genomes of European ancestry reference panel using the LD matrix of the LDLink program. We observed three LD blocks with 3 top-ranked SNPs with $p<5x10^{-8}$ (red box on SNP).

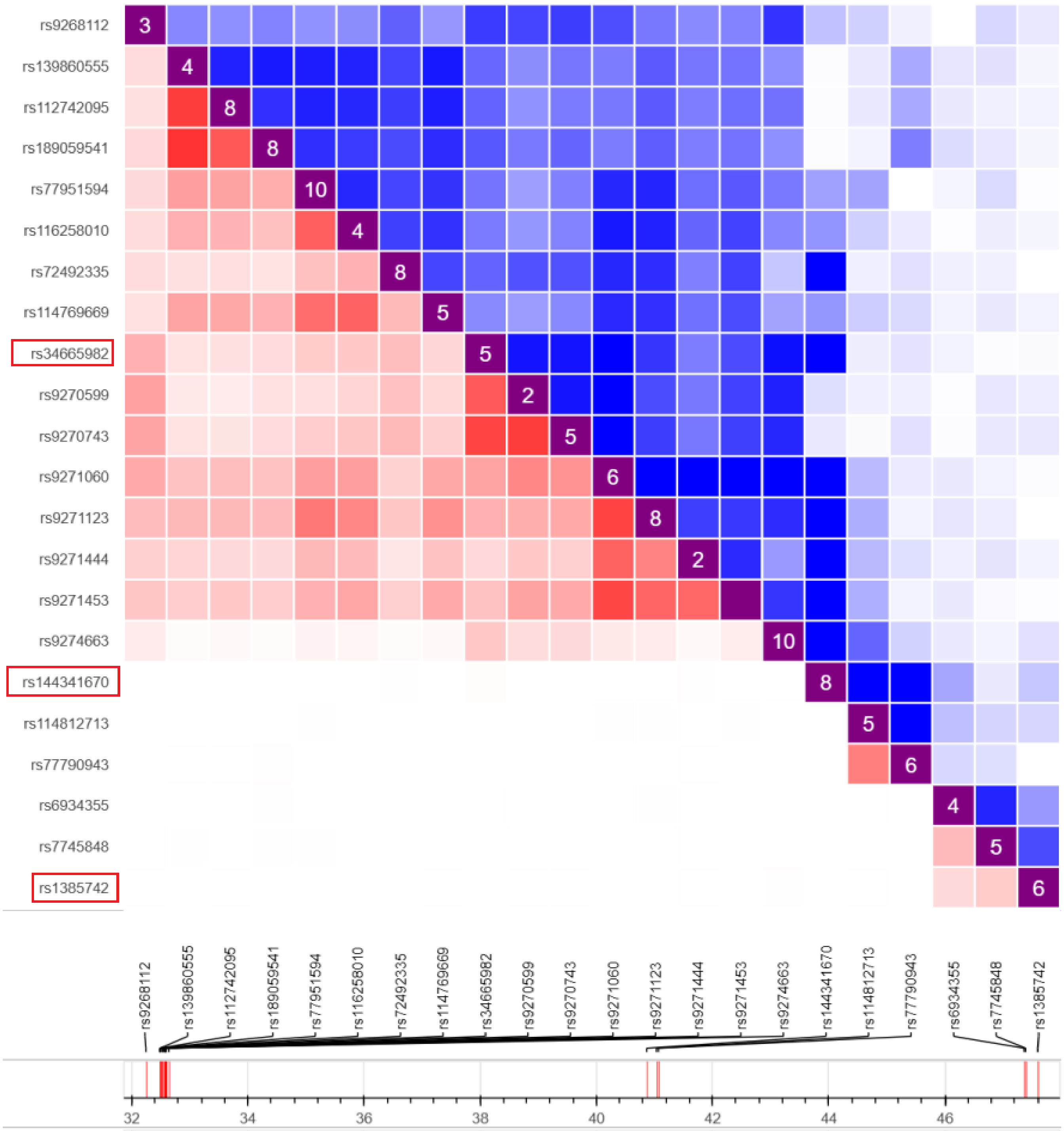

Supplement: Supplementary file 1 [file jcm-15-02885-s001.zip › jcm-4151532-supplementary.pdf]
